# Band‐Aid‐Like Self‐Fixed Barrier Membranes Enable Superior Bone Augmentation

**DOI:** 10.1002/advs.202206981

**Published:** 2023-04-08

**Authors:** Qianqian Li, Wenyi He, Weiran Li, Shulu Luo, Minghong Zhou, Dingcai Wu, Yan Li, Shuyi Wu

**Affiliations:** ^1^ Hospital of Stomatology Guanghua School of Stomatology Guangdong Provincial Key Laboratory of Stomatology Sun Yat‐sen University Guangzhou 510055 P. R. China; ^2^ PCFM Lab School of Chemistry Sun Yat‐sen University Guangzhou 510006 P. R. China; ^3^ Medical Research Institute Guangdong Provincial People's Hospital (Guangdong Academy of Medical Sciences) Southern Medical University Guangzhou 510080 P. R. China

**Keywords:** bone defect treatment, bone graft material immobilization, guided bone regeneration, self‐fixed barrier membrane, suction‐adhesion

## Abstract

In guided bone regeneration surgery, a barrier membrane is usually used to inhibit soft tissue from interfering with osteogenesis. However, current barrier membranes usually fail to resist the impact of external forces on bone‐augmented region, thus causing severe displacement of membranes and their underlying bone graft materials, eventually leading to unsatisfied bone augmentation. Herein, a new class of local double‐layered adhesive barrier membranes (ABMs) is developed to successfully immobilize bone graft materials. The air‐dried adhesive hydrogel layers with suction‐adhesion properties enable ABMs to firmly adhere to the wet bone surface through a “stick‐and‐use” band‐aid‐like strategy and effectively prevent the displacement of membranes and the leakage of bone grafts in uncontained bone defect treatment. Furthermore, the strategy is versatile for preparing diverse adhesive barrier membranes and immobilizing different bone graft materials for various surgical regions. By establishing such a continuous barrier for the bone graft material, this strategy may open a novel avenue for designing the next‐generation barrier membranes.

## Graphical abstract

Local double‐layered adhesive barrier membranes with suction‐adhesion properties can firmly adhere to the wet bone surface through a “stick‐and‐use” band‐aid‐like strategy and effectively prevent the displacement of membrane and the leakage of bone graft materials, leading to superior bone augmentation in guided bone regeneration.

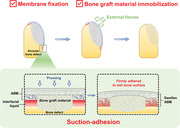

## 1 Introduction

Nowadays, dental implants have been widely considered as the optimum treatment option for the functional and aesthetic restoration of missing teeth.^[^ ^1^ ^]^ The prerequisite for ideal implant placement and good long‐term prognosis is that there is sufficient bone volume in the edentulous ridge to accommodate the implant.^[^ ^2^ ^]^ However, insufficient alveolar bone height and/or width caused by tooth loss, periodontitis, trauma, and other reasons are commonly encountered in clinical practice.^[^ ^3^ ^]^ More than 50% of implant placements need to be combined with bone augmentation surgeries to achieve good functional reconstructions and aesthetic effects.^[^ ^1^, ^4^ ^]^

**Figure 1:**
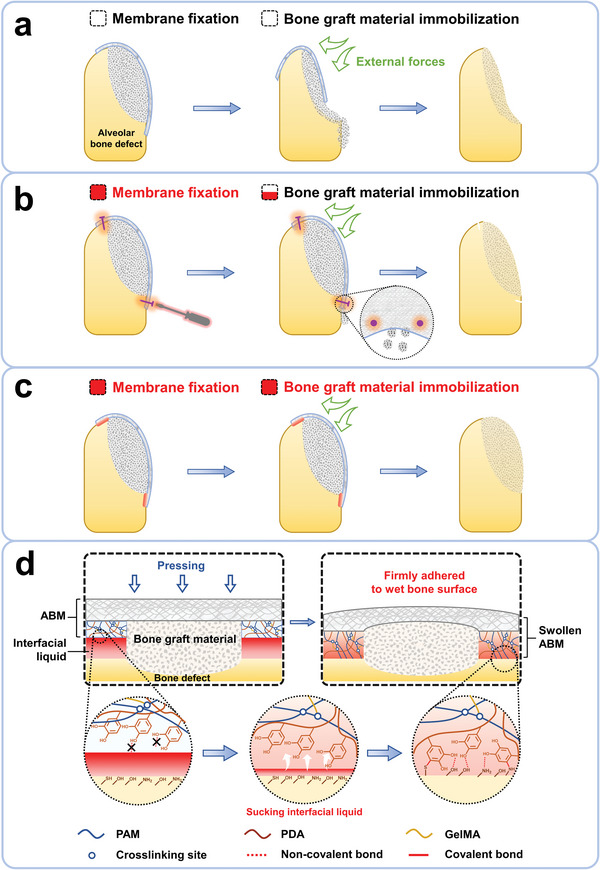
Schematic illustration of bone augmentation through GBR with commercial barrier membrane, commercial barrier membrane fixed with pins, and ABM, as well as the adhesive mechanism of ABM toward the wet bone surface. a) Commercial barrier membrane utilized in GBR surgery can prevent soft tissue from growing into bone defect region, but external forces can usually cause displacement of membrane and its underlying bone graft material, resulting in unsatisfied bone augmentation. b) Barrier membrane discontinuously fixed with pins can reduce the displacement of bone graft material but may still fail to avoid the lateral and apical leakage of material. c) With outstanding self‐fixation and bone graft material immobilization effects, ABM can establish a continuous barrier for the bone graft material and lead to a satisfactory bone regeneration effect. d) ABM with a DAH layer can effectively suck interfacial liquid and form both non‐covalent and covalent bonds with bone surface, eventually leading to its high adhesion strength to the bone.

Guided bone regeneration (GBR) is the most widely used bone augmentation surgery in implantology currently,^[^ ^5^ ^]^ which uses a barrier membrane to prevent soft tissue from growing into bone defect region.^[^ ^6^ ^]^ During the process of wound closure and healing, external forces on the bone‐augmented region, such as the forces generated in flap closure, movement of adjacent muscles, and chewing food, can cause significant displacement of barrier membranes (e.g., collagen membranes) and underlying bone graft materials (e.g., particulate bone grafts), which will seriously affect the quality and quantity of bone augmentation (**Figure** **1**a).^[^ ^5^, ^7^ ^]^ Therefore, how to limit the bone graft material to the desired position while fixing the membrane is extremely important for the success of GBR.^[^ ^8^ ^]^ To address this issue, pins are usually applied in clinical practice to discontinuously fix the barrier membrane. This method, to a certain extent, can reduce the displacement of the particulate bone graft materials,^[^ ^5^, ^7^ ^]^ but still cannot avoid the lateral and apical leakage of materials from the space between pins (Figure 1b). For uncontained bone defectsor severe bone defects, block bone grafts fixed with screws are often used to maintain the spatial stability of the bone‐augmented regions.^[^ ^2^, ^9^ ^]^ Nevertheless, the use of any pins or screws increases the risks of damaging the alveolar nerve, the root of adjacent teeth, and other important anatomical structures, and easily causes a bone fracture in the bone block or receipt region.^[^ ^7^, ^10^ ^]^ Therefore, there is an urgent need for a convenient and effective self‐fixation membrane in bone augmentation surgery to spatiotemporally provide bone graftmaterial with excellent full‐scale barrier effects such as displacement prevention and leakage prevention.^[^ ^10^, ^11^ ^]^

Herein, a new class of local double‐layered adhesive barrier membranes (ABMs) is successfully developed by integrating commercial collagen membranes (CMs) with polyacrylamide/polydopamine (PAM/PDA) adhesive hydrogels and air‐drying, and their excellent osteogenesis effect in bone augmentation surgery is demonstrated (Figure 1c). For the as‐constructed ABMs, the dried adhesive hydrogel (DAH) layer with suction‐adhesion properties can rapidly suck liquid on the bone surface which may interfere the adhesion (such as blood and saliva) in the mouth, and firmly adhere to the bone surface through abundant catechol groups of PDA. After sucking liquid on the bone surface, the hydrogels quickly swell and become flexible, and thus they can adapt to various irregular bone surfaces and establish a continuous and seamless barrier for bone graft spatially (Figure 1d). The mechanical and liquid‐sucking properties of the hydrogels can be tuned on demand by adjusting the composition of the crosslinkers and the resulting ABMs can be utilized for immobilizing various bone graft materials (e.g., particulate and block bone grafts) to meet the requirements of different surgical regions. In brief, ABMs with high adhesive strength and tunable physicochemical properties can effectively prevent the displacement of GBR membrane and the leakage of bone graft materials through a “stick‐and‐use” band‐aid‐like strategy, thus establishing a continuous barrier for the bone graft material and eventually leading to satisfactory bone regeneration effect in vivo. We hope that our adhesive barrier membrane will become a promising and versatile material for clinical bone augmentation surgery.

## 2 Results and Discussion

**Figure 2:**
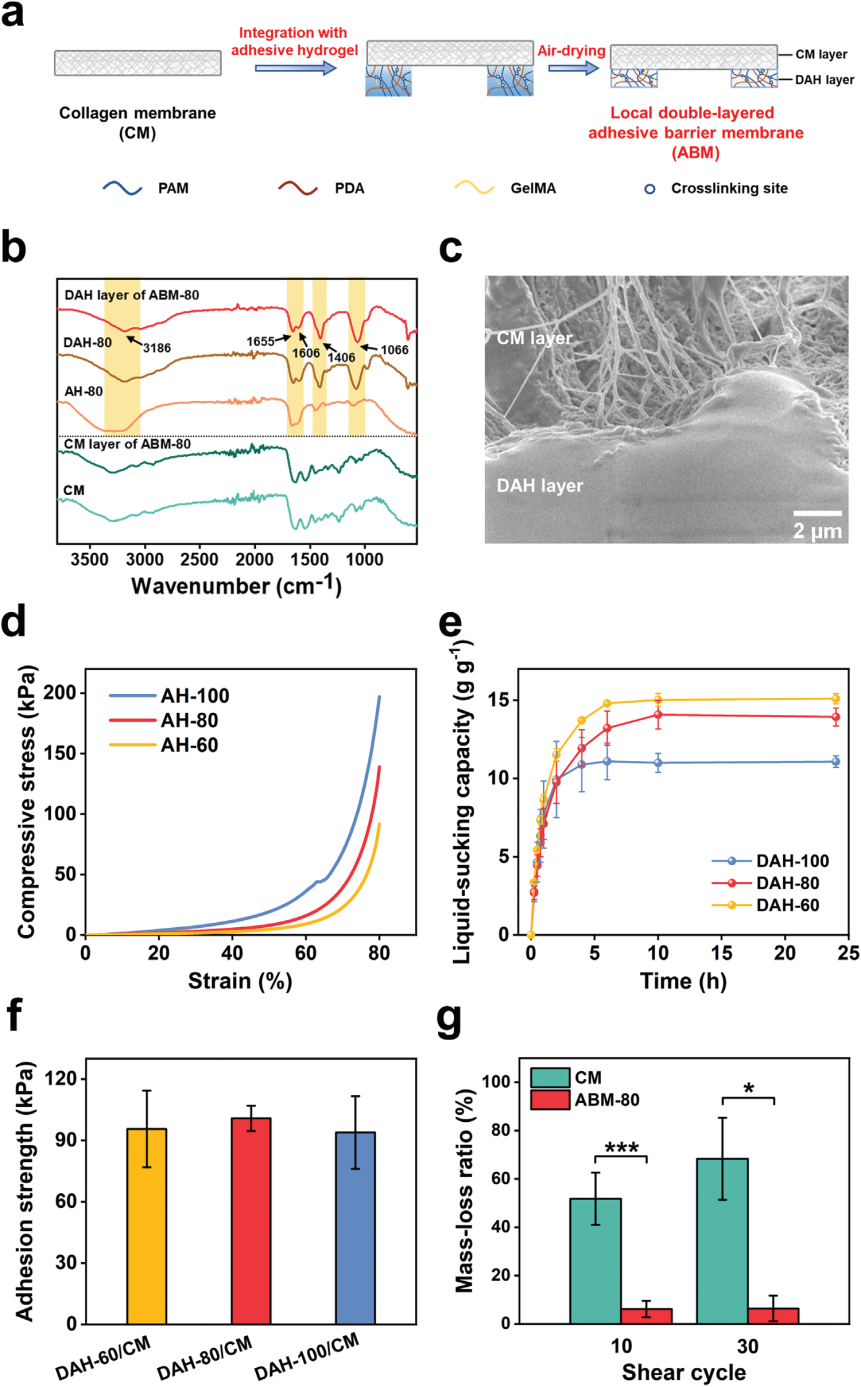
a) Schematic illustration for the preparation of ABM. b) FTIR spectra of AH‐80, DAH‐80, DAH layer of ABM‐80, CM, and CM layer of ABM‐80. c) Cross‐sectional SEM image of ABM‐80. d) Compressive stress‐strain curves of AH‐60, AH‐80, and AH‐100. e) Liquid‐sucking capacity‐time curves of DAH‐60, DAH‐80, and DAH‐100 immersed in PBS (*n* = 4). f) Adhesion strengths of DAH‐60/CM, DAH‐80/CM, and DAH‐100/CM (*n* = 3). g) Mass‐loss ratios of CM and ABM‐80 after 10 and 30 shear cycles (*n* = 4; Student's *t‐*test for 10 cycles, Mann–Whitney *U* test for 30 cycles; * *p* < 0.05, *** *p* < 0.001).All error bars = SD.

The preparation of our ABM includes the synthesis of PAM/PDA adhesive hydrogel (Figures S1 and S2, Supporting Information), integration of adhesive hydrogel and CM for constructing local double‐layered membrane, and air‐drying in blast drying oven (**Figure** **2**a and Figure S3, Supporting Information). PAM/PDA adhesive hydrogel is synthesized by referring to our previous method,^[^ ^12^ ^]^ except for replacing *N*,*N*’‐methylenebisacrylamide (MBAA) crosslinker with MBAA and methacrylate gelatin (GelMA) crosslinkers. Dopamine is first pre‐polymerized with ammonium persulfate under stirring at room temperature for 30 min, and then acrylamide monomer, MBAA and GelMA crosslinkers, and tetramethylethylenediamine accelerator are added into the reaction system to undergo the gelation at 60 °C for 12 h, leading to the formation of PAM/PDA adhesive hydrogel. For the adhesive hydrogel, the PDA component with abundant catechol groups is used for tissue adhesive^[^ ^13^ ^]^ and the PAM component acts as the hydrogel scaffold.^[^ ^14^ ^]^ The as‐obtained ABM consists of a dried PAM/PDA adhesive hydrogel layer (air‐drying at 40 °C overnight) and a CM layer. As shown in Fourier transform infrared (FTIR) spectrum (Figure 2b) of the DAH layer of ABM with a *W*_MBAA_/*W*_MBAA+GelMA_ ratio (denoted as MBAA ratio) of 80 wt% (ABM‐80), the board peak at ∼3186 cm^−1^ is ascribed to O–H stretching of PDA and N‐H stretching of PAM and PDA;^[^ ^15^ ^]^ the peak at 1655 cm^−1^ is C=O stretching of PAM; N–H bending vibration peak at 1606 cm^−1^, C–N stretching vibration peak at 1406 cm^−1^, and ‐NH_2_ in‐plane rocking peak at 1066 cm^−1^ are from both PAM and PDA.^[^ ^16^ ^]^ As shown in Figure 2b, an adhesive hydrogel with an MBAA ratio of 80 wt% (AH‐80) and dried AH‐80 (DAH‐80) have similar FTIR spectra to the DAH layer of ABM‐80 because all of them have PDA and PAM components. The CM layer of ABM‐80 and CM show very similar FTIR spectra (Figure 2b); the CM layer of ABM‐80 exhibits an asymmetric porous structure (Figure S4, Supporting Information), which is similar to that of the reported CM.^[^ ^17^ ^]^ These results indicate that air‐drying in a blast drying oven at 40 °C overnight does not affect CM's chemical structure and morphology. Moreover, as shown in the SEM image of Figure 2c, ABM‐80 exhibits a tightly integrated interface of the DAH and CM layers.

In order to match the requirements for different commercial membranes or surgical regions, adhesive hydrogels with different MBAA ratios are prepared. MBAA and GelMA crosslinkers can form covalent crosslinking sites in the PAM hydrogel framework to tune hydrogel's properties. Adhesive hydrogels with MBAA ratios of 60 and 100 wt% are denoted as AH‐60 and AH‐100, respectively (Table S1, Supporting Information). As the adhesive layer of the barrier membrane, adhesive hydrogels should have enough compressive strength to withstand external forces.^[^ ^18^ ^]^ As shown in Figure 2d, compressive strengths at 80% strain of AH‐60, AH‐80, and AH‐100 are as high as 92, 139, and 197 kPa, respectively. Such a reinforcement tendency is attributed to the higher crosslinking densities provided by higher MBAA ratios.^[^ ^18c^ ^]^ An ideal adhesive hydrogel for GBR should tolerate the cyclic compressive stress for maintaining space for osteogenesis. After 20 cycles of loading‐unloading test, all the adhesive hydrogels can basically recover to their initial shape under the stress of 10 kPa (Figure S5, Supporting Information), indicating their good recoverability. Furthermore, AH‐80 exhibits 383% tensile strain at break (Figure S6, Supporting Information), which is significantly higher than the commercial CM,^[^ ^6b^ ^]^ indicating that our adhesive hydrogel has superior mechanical properties.

Generally, the intraoral bone‐augmented region is a dynamic and wet environment with blood and saliva, and the interfacial liquid may severely weaken the adhesive strength of hydrogel to the bone surface during surgery. In order to efficiently remove interfacial liquid, the as‐constructed double‐layered membrane with adhesive hydrogels and CM was air‐dried to endow adhesive ability in wet environments in vivo. Dried AH‐60 and AH‐100 are denoted as DAH‐60 and DAH‐100, respectively. As illustrated in Figure 2e, the liquid‐sucking capacities of DAH‐60, DAH‐80, and DAH‐100 after immersion in phosphate‐buffered saline (PBS) solution for 15 min are 3.4, 2.7, and 2.8 g g^−1^, respectively, indicating that they can dry the wet bone surface rapidly. After 10 h, they reach an equilibrium state, with liquid‐sucking capacities of 15.0, 14.1, and 11.0 g g^−1^, respectively. The lower equilibrium liquid‐sucking capacities of DAH‐80 and DAH‐100 are ascribed to their higher crosslinking densities from the higher MBAA ratios.^[^ ^18c^ ^]^ These results suggest that DAHs with tunable and rapid liquid‐sucking characteristics might meet the requirements for sucking interfacial liquid in different surgical regions.

We evaluate the adhesion strength of DAHs and the as‐constructed ABMs to wet bone surfaces in vitro. As displayed in Movie S1, Supporting Information, DAH‐80 can firmly adhere to a wet bone and easily lift 0.6 kg after pressing gently for about 40 s. In sharp contrast, undried AH‐80, which belongs to a conventional PDA hydrogel, can hardly adhere to the wet bone surface due to the interference of interfacial liquid (Movie S2, Supporting Information). These results indicate that drying plays a key role in the preparation of our ABM with superb adhesion properties to wet bone surface, which is the difference between our DAHs and the conventional PDA hydrogels. To test the adhesion performance of ABMs, a lap‐shear test between a moist bone and a membrane composed of DAH and CM (DAH/CM) is conducted (Figure S7, Supporting Information). DAH‐60/CM, DAH‐80/CM, and DAH‐100/CM exhibit high adhesion strength of 96, 101, and 94 kPa, respectively (Figure 2f). The as‐mentioned strong tissue adhesion is attributed to rapid sucking ability and catechol‐mediated interfacial interactions of DAHs. When DAH/CM contact with the wet bone surface, DAH sucks interfacial liquid rapidly and dries the wet surface.^[^ ^19^ ^]^ Simultaneously, the catechol groups on hydrogel form hydrogen bonds and covalent bonds (e.g., Schiff base or Michael addition reaction) with bone surface,^[^ ^13^, ^20^ ^]^ resulting in high adhesion strength. Thus, we speculate that ABMs with good adhesiveness may effectively immobilize barrier membranes and bone graft materials. To simulate the external forces on the augmented region, we perform a shear friction test between the periosteum and bone to evaluate the ability of ABM to resist cyclic shear friction and prevent leakage of bone grafts in vitro (Figures S8–S10, Supporting Information). After 10 cyclic shearings, only 6.1% of bone grafts for the ABM‐80 group are displaced, as sharply compared to 51.8% of the CM group. No further obvious leakage of bone grafts for the ABM‐80 group (6.4%) is measured after 30 shear cycles, significantly lower than the CM group (68.4%) (Figure 2g). The results indicate that the wet adhesive ability endows ABMs with outstanding self‐fixation and bone graft material immobilization effects in vitro.

**Figure 3:**
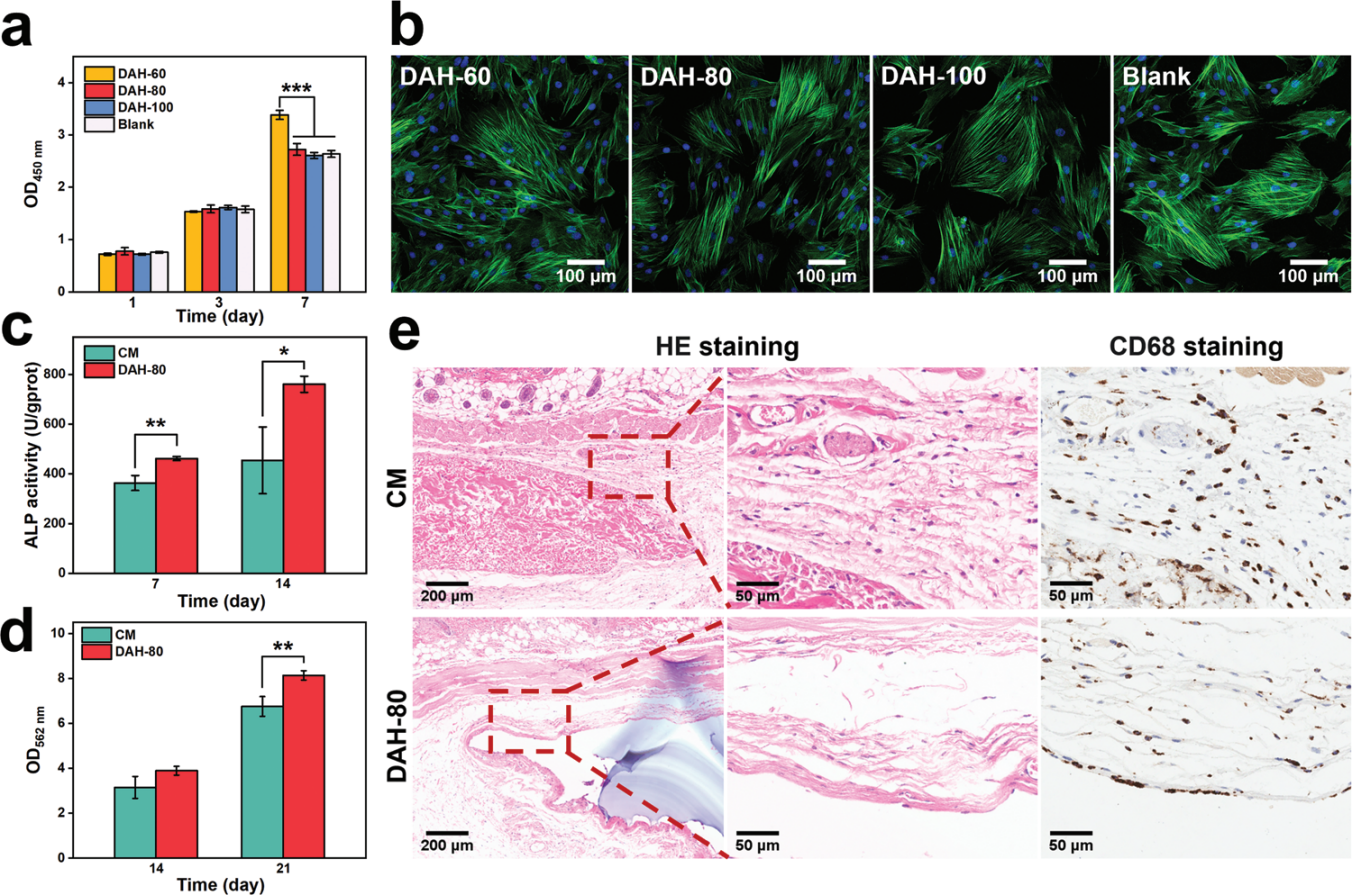
a) CCK‐8 assay results of BMSCs cultured in conditioned culture mediums of DAHs for 1, 3, and 7 days (*n* = 3; Kruskal–Wallis's nonparametric test for the 1^st^ day, one‐way ANOVA followed by LSD test for the 3^rd^ and 7^th^ days). b) Morphology of BMSCs cultured in conditioned culture mediums of DAHs for 3 days (green for F‐actin and blue for cell nucleus). c) ALP activity of BMSCs cocultured with CM or DAH‐80 in osteogenic induction medium for 7 and 14 days (*n* = 3; Student's *t‐*test). d) Semi‐quantitative analysis of calcium deposits following ARS staining after BMSCs are cocultured with CM or DAH‐80 in osteogenic induction medium for 14 and 21 days (*n* = 3; Student's *t‐*test). e) HE staining and CD68 immunohistochemical staining of tissues around CM or DAH‐80 embedded in the backs of nude mice for 28 days. * p < 0.05, ** p < 0.01, *** p < 0.001; all error bars = SD.

We further evaluate the biological properties of our DAHs. Before the biological characterization, our samples are treated with purification by deionized water and sterilization by ethylene oxide. First of all, we evaluate the biological safety of DAHs by measuring their cytocompatibility and histocompatibility. Cell Counting Kit‐8 (CCK‐8) results (**Figure** **3**a) show that the cell viability of DAH‐60, DAH‐80, DAH‐100, and blank control groups increases with time and there is no significant difference between DAH groups and the blank control group on the 1^st^ and 3^rd^ days, indicating that our samples have no adverse effects on bone marrow mesenchymal stem cells (BMSCs) proliferation. The cell activity of the DAH‐60 group is significantly higher than that of other groups on the 7^th^ day, probably because its culture medium containing more degraded peptides of GelMA improves the cell proliferation activity. The fluorescence staining results (Figure 3b) show that the BMSCs of each group are fully extended and their cytoskeletons appear in a filamentous arrangement in the same direction. At the same time, the material should not affect osteogenic differentiation. DAH‐80 is selected as a representative sample to examine the effect on the osteogenic differentiation ability of BMSCs. We measure alkaline phosphatase (ALP) expression as an indicator of early osteogenesis and use Alizarin Red S (ARS) staining to analyze late calcium deposition.^[^ ^21^ ^]^ After osteogenic induction of BMSCs for 7 and 14 days, ALP activity in DAH‐80 and CM groups increases with time, and the DAH‐80 group presents higher activity (Figure 3c). In addition, ARS staining of BMSCs following osteogenic induction for 21 days exhibits that there are significantly more calcium nodules in the DAH‐80 group, and the semi‐quantitative results show the same trend (Figure 3d and Figure S11, Supporting Information). We speculate that GelMA in DAHs could be degraded into the culture medium during coculture. The degraded peptides can be conducive to cell adhesion, migration, and proliferation, making DAH‐80 a promoting effect on cell proliferation and osteogenic differentiation.^[^ ^22^ ^]^

To assess the histocompatibility of our DAHs, DAH‐80, and FDA‐approved CM are implanted into the backs of nude mice for 14 and 28 days. All wounds heal well without purulent secretion or other obvious inflammations. The materials and surrounding tissues are taken out for histological staining. It can be seen in HE staining that some inflammatory cells infiltrate around the materials in both groups after implanting for 14 days (Figure S12, Supporting Information) and synchronously reduce after 28 days (Figure 3e). Immunohistochemical staining for the macrophage marker CD68 shows that there is no significant difference in the distribution and quantity of macrophages between DAH‐80 and CM groups. The results indicate that our DAH‐80 shows good biocompatibility in vitro and in vivo, and the as‐constructed ABM‐80 consisting of the CM and DAH layers would have similar yet excellent biocompatibility.

**Figure 4:**
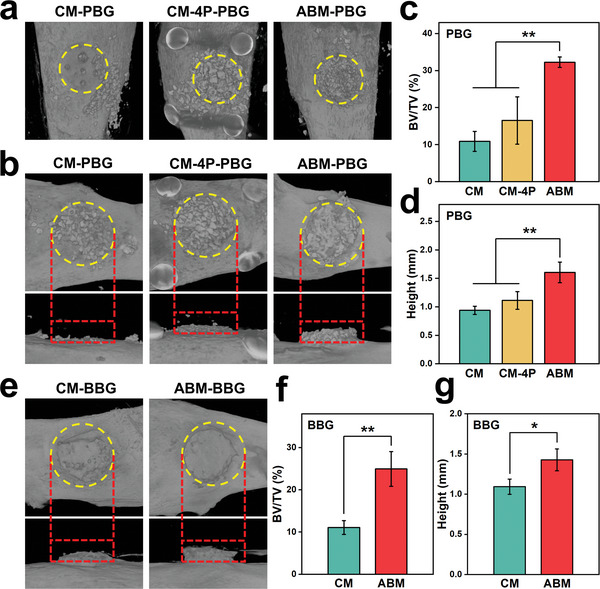
a) Representative images of micro‐CT 3D reconstructions of uncontained bone defects in rabbit tibias after placing PBG and covering with CM, CM‐4P, or ABM for 10 days. b) Representative images of micro‐CT 3D reconstructions of augmented regions of different groups after filling PBG for 2 months. c) BV/TV and d) height of augmented bone of different groups after filling PBG for 2 months (*n* = 3; one‐way ANOVA followed by LSD test). e) Representative images of micro‐CT 3D reconstructions of augmented regions of different groups after filling BBG for 2 months. f) BV/TV and g) height of augmented bone of different groups after filling BBG for 2 months (*n* = 3; Student's *t‐*test). Bone defect region is identified by a yellow circle and the height of augmented bone is identified by a red rectangle; * p < 0.05, ** p < 0.01; all error bars = SD.

The excellent performances of DAH‐80 promote us to construct ABM‐80 and explore its ability to limit the graft materials orthotopically in vivo. At present, most animal models of bone defects involving barrier membranes are calvarial defect models.^[^ ^23^ ^]^ However, these models are difficult to reflect the influence of complex oral activities on barrier membranes and bone graft materials in the bone‐augmented region, because of the limited mobility of the soft tissue above the skull in the daily life of animals. In order to simulate the displacement of membrane and bone graft material caused by suturing and subsequent movements, a more appropriate bone defect model needs to be established. By observing the activities of rabbits, we find that the knee joint is one of the most frequently moved joints. Daily activities such as squatting, standing, and turning cause knee bending, stretching, and internal and external rotations, which may result in obvious relative movement between the soft tissues and the medial proximal tibias (Figure S13, Supporting Information). Therefore, we believe that the medial proximal tibia is an appropriate place to test the immobilization effect of bone graft materials. We decorticate the medial proximal tibias of rabbits to form uncontained bone defects and place heterogenous particulate bone graft (PBG) or autogenous block bone graft (BBG). Subsequently, we cover the bone graft materials with ABM in the experimental group(where ABM‐80 is chosen as a representative sample of ABMs) and with CM in the negative control group (Figure S14, Supporting Information). As for the positive control group, PBG is covered with CM, which is then fixed by four titanium pins (CM‐4P). As shown in micro‐CT scanning results (**Figure** **4**a), only a small amount of PBG is left in the defect region in the CM group after 10 days of surgery. In sharp contrast, ABM can retain almost all the PBG even in this harsh simulated environment, and its retention amount is even higher than that of the CM‐4P group. These results indicate that ABM has a good ability to prevent the displacement and leakage of bone graft materials in the early stage of osteogenesis. At 2 months after filling PBG, the highest augmented bone volume fraction (bone volume/total volume, BV/TV; 32%) and augmented bone height (1.61 mm) are observed in the ABM group, as compared to the CM group (11%, 0.94 mm) and even the CM‐4P group (17%, 1.11 mm) (Figure 4b–d). It is worth pointing out that these pins have been firmly integrated with bones at 2 months after surgery and are extremely difficult to remove. This situation can also be encountered in clinical practice but can be avoided if ABM is used. Furthermore, when the defect regions are filled with BBG, the ABM group can also achieve remarkably better bone augmentation effect, in which BV/TV (25%) and height (1.43 mm) of augmented bone are much higher than those of the CM group (11%, 1.09 mm) at 2 months after surgery (Figure 4e–g). The results could be explained as follows: at the early stage, the high adhesive strength of ABM could fix the bone graft materials to the defect region to prevent their displacement and leakage; at the later stage, the hydrogel could swell restrictedly under the sutured flap and thus establish a three‐dimensional barrier to the graft materials, eventually forming a satisfied bone volume in the desired region.

**Figure 5:**
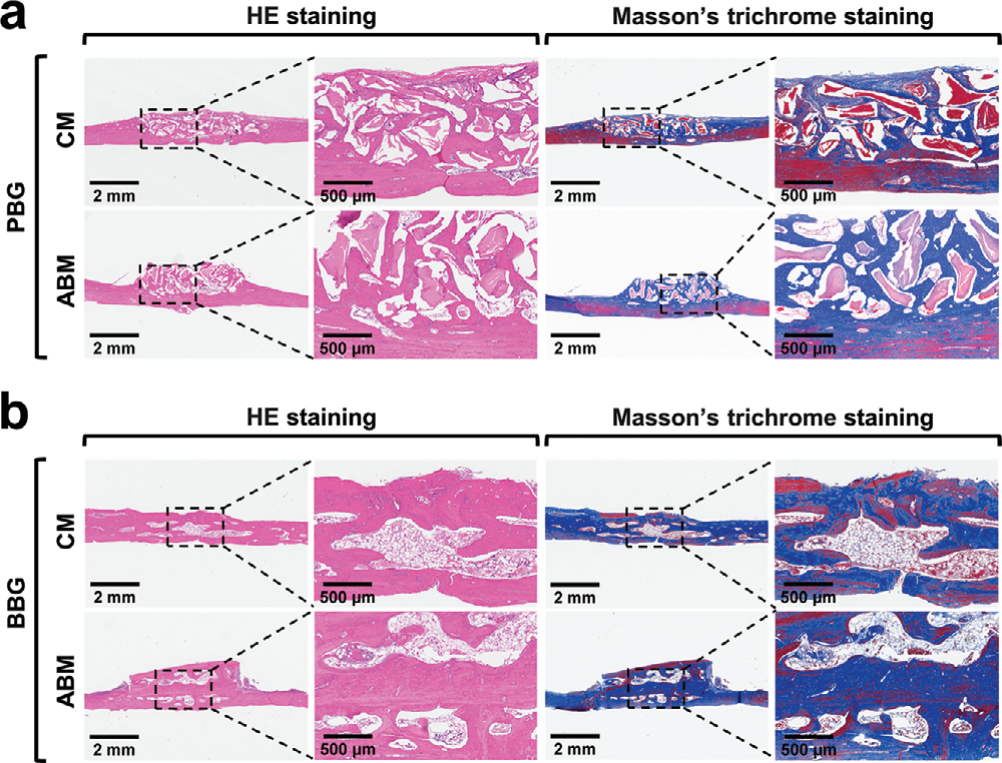
HE staining and Masson's trichrome staining of the new bones augmented in defect regions at 2 months after placing a) PBG and b) BBG, followed by covering with CM or ABM.

HE staining results show that at 2 months after surgery, the new bone volume and density are significantly better in the ABM group with PBG than in the CM group with PBG (**Figure** **5**a). After filling BBG, a good bony connection can be formed between the grafted bone block and cortex of defect region in the tibia in the ABM group, leading to an excellent bone augmentation effect, while the bone block in the CM group is severely resorbed (Figure 5b). Masson's trichrome staining results further demonstrate that the new bone is more continuous, denser, and thicker in the ABM group. The histological results are consistent with the micro‐CT results. Taken together, ABM can retain much more bone graft materials in the bone‐augmented region, eventually forming an adequate bone volume and bone height.

## 3 Conclusion

We afford a facile strategy for constructing a new class of local double‐layered adhesive barrier membranes by synthesizing adhesive hydrogels, integrating with commercial CMs, and air‐drying. With unique suction‐adhesion properties, our ABM can rapidly suck interfacial liquid and tightly adhere to the wet bone surface. Through the “stick‐and‐use” band‐aid‐like strategy, ABM exhibits outstanding self‐fixation and bone graft material immobilization effects in uncontained bone defect treatments, leading to a superior regeneration effect to CM. Furthermore, our ABMs can be used to fix a variety of bone graft materials to meet various clinical needs. In conclusion, our ABMs show a promising application prospect for bone augmentation surgery, and hopefully, our strategy can provide a new direction for designing the next‐generation of barrier membranes.

## 4 Experimental Section

The Experimental Section is available in the Supporting Information.

## Conflict of Interest

The authors declare no conflict of interest.

## Supporting information

Supporting Information

Supplemental Movie 1

Supplemental Movie 2

## Data Availability

The data that support the findings of this study are available in the supplementary material of this article.
