# Supplementary material for: Band‐Aid‐Like Self‐Fixed Barrier Membranes Enable Superior Bone Augmentation
Source: Adv Sci (Weinh). 2023 Apr 8;10(16):2206981. doi: 10.1002/advs.202206981 (PMC10238180; doi:10.1002/advs.202206981)
Supplement: Supplementary file 1 — Supporting Information [file ADVS-10-2206981-s003.pdf]

ADVANCED SCIENCE
Open Access

# Supporting Information

for *Adv. Sci.*, DOI 10.1002/advs.202206981

Band-Aid-Like Self-Fixed Barrier Membranes Enable Superior Bone Augmentation

*Qianqian Li, Wenyi He, Weiran Li, Shulu Luo, Minghong Zhou, Dingcai Wu*, Yan Li* and Shuyi Wu**

ADVANCED SCIENCE
Open Access

# Supporting Information

for *Adv. Sci.*, DOI 10.1002/advs.202206981

Band-Aid-Like Self-Fixed Barrier Membranes Enable Superior Bone Augmentation

*Qianqian Li, Wenyi He, Weiran Li, Shulu Luo, Minghong Zhou, Dingcai Wu*, Yan Li* and Shuyi Wu**

# Supporting Information

**Band-Aid-Like Self-Fixed Barrier Membranes Enable Superior Bone Augmentation**

*Qianqian Li, Wenyi He, Weiran Li, Shulu Luo, Minghong Zhou, Dingcai Wu*, Yan Li* and Shuyi Wu**

Q. Li, W. Li, S. Luo, Y. Li, S. Wu
Hospital of Stomatology,
Guanghua School of Stomatology,
Guangdong Provincial Key Laboratory of Stomatology,
Sun Yat-sen University,
Guangzhou 510055, P. R. China
E-mail: liy8@mail.sysu.edu.cn
E-mail: wushuyi@mail.sysu.edu.cn

W. He, D. Wu
PCFM Lab,
School of Chemistry,
Sun Yat-sen University,
Guangzhou 510006, P. R. China.
E-mail: wudc@mail.sysu.edu.cn

M. Zhou
Medical Research Institute,
Guangdong Provincial People's Hospital (Guangdong Academy of Medical Sciences),
Southern Medical University,
Guangzhou 510080, P. R. China.

***Materials***

Dopamine hydrochloride (DA), acrylamide (AM), *N,N'*-methylenebisacrylamide (MBAA) and tetramethylethylenediamine (TEMDA) were purchased from Macklin (China). Ammonium persulfate (APS) was purchased from J&K Scientific (China). Methacrylate gelatin (GelMA; MA degree 97%) was purchased from Aladdin (China). Bone marrow mesenchymal stem cells (BMSCs) were isolated from 2-week-old Sprague-Dawley (SD) male rats. Dulbecco's modified eagle medium/nutrient mixture F-12 (DMEM/F-12), fetal bovine serum (FBS) and phosphate-buffered saline (PBS; 0.01 M, pH 7.4) solution were purchased from Gibco (America). Cell Counting Kit-8 (CCK-8) was purchased from Dojindo (Japan). Collagen membrane (Bio-Gide) and heterogenous particulate bone graft (Bio-Oss) were purchased from Geistlich (Switzerland). 4',6-diamidino-2-phenylindole (DAPI) and Actin-Tracker Green were purchased from Beyotime (China). Falcon cell culture insert was purchased from Corning (America). Alkaline phosphatase (ALP) Assay Kit was purchased from Jiancheng (China). Alizarin Red S (ARS) staining solution was purchased from Cyagen (China). Titanium pin was purchased from Trausim (China). Trephine drill, round drill, and pioneer drill were purchased from öko Dent (Germany). EDTA decalcifying solution was purchased from Servicebio (China). Others were used as received.

***Preparation of adhesive hydrogel***

The adhesive hydrogel was prepared as follows: DA was dissolved in deionized water; subsequently, APS was added and the solution was stirred at room temperature; after 30 min, AM, MBAA, GelMA and TEMDA were then added into the solution. The resulting solution was poured into molds and heated in oven at 60 °C for 12 h for gelation, leading to formation of adhesive hydrogel. For various adhesive hydrogel samples, the dosage of their raw materials was listed in Table S1.

***Preparation of dried adhesive hydrogel (DAH)***

Our adhesive hydrogel was air-dried in blast drying oven at 40 °C overnight to obtain DAH.

***Preparation of local double-layered adhesive barrier membrane (ABM)***

Collagen membrane (CM) was cut into rectangle-shaped samples (10 mm × 10 mm). Adhesive hydrogel was cut into rectangle-shaped samples (10 mm × 10 mm) with a circle hole

in the center (6 mm diameter). The loose bottom-surface of CM was integrated with adhesive hydrogel and air-dried in blast drying oven at 40 °C overnight to obtain the finial ABM.

***Material characterization***

*Fourier transform infrared (FTIR) spectroscopy analysis.* Samples' functional groups were characterized by FTIR spectrometer (Nicolet iS50, Thermo scientific, America) with an attenuated total reflectance attachment and with a total of 32 scans.

*Mechanical test.* All mechanical tests were performed on a universal mechanical testing machine (WD-5A, Guangzhou Experimental Instrument Factory, China) at room temperature. For compressive tests, the cylinder-shaped adhesive hydrogels (21 mm diameter and 9 mm height) were compressed at the speed of 2 mm $min^{-1}$. The cyclic compressive tests were characterized by 20 cyclic loading-unloading compressive cycles by reaching 10 kPa compressive stress at the speed of 2 mm $min^{-1}$. For tensile tests, adhesive hydrogels were cut into dumbbell-shaped pieces (50 mm length and 3 mm width) and stretched at the speed of 30 mm $min^{-1}$.

*Measurement of liquid-sucking capacity.* The initial weight of DAHs was measured as $w_0$. DAHs were then immersed into PBS solution in the 37 °C water bath. At predetermined time, the weight of the swollen hydrogels was measured as $w_t$. The liquid-sucking capacity was calculated according to equation (1):

$$\text{Liquid-sucking capacity} = \frac{w_t - w_0}{w_0} \qquad (1)$$

*Scanning electron microscopy (SEM) observation.* The ABM-80 was frozen in liquid nitrogen for 5 h. Its DAH layer was crushed and its CM layer was cut with scalpel in liquid nitrogen. After freeze-drying for 1 day, its surface and interface were investigated by SEM (Sigma 300, Zeiss, Germany).

*Adhesion test.* Adhesion tests were performed on a universal mechanical testing machine (WD-5A, Guangzhou Experimental Instrument Factory, China) at room temperature. The loose surface of CM (20 mm length and 10 mm width) was integrated with adhesive hydrogel (10 mm × 10 mm) and air-dried in blast drying oven at 40 °C to obtain DAH/CM. Moreover, a bone was stuck onto a piece of plastic plate. During the test, the DAH/CM was adhered on the moist bone by applying a pressure of ≈ 170 kPa for 2 min. The ends of CM and plastic plate were clamped separately and pulled upward at a speed of 1 mm $min^{-1}$. The adhesions of both bone-swollen adhesive hydrogel and CM-swollen adhesive hydrogel interfaces were simultaneously measured by lap-shear test. The adhesion strength was calculated by dividing the maximum stress by the initial adhesion area.

*Measurement of bone graft material leakage in vitro*. The measurement of leakage was performed on a coefficient of friction tester (MS-MX-D, Guangdong Rongguang, China) at room temperature. Before examination, the bone and periosteum around the bone were separated. The bone with a circular bone defect (6 mm diameter) created by a trephine drill and a round drill was glued onto the plane and moistened, and the periosteum was glued onto the sled. Heterogenous particulate bone graft (PBG) with an initial weight of $m_0$ (≈ 16 mg) was filled in the circular bone defect. Subsequently, CM or ABM-80 was covered onto the adjacent bone surface of the defect. During the test, the drive speed was set at 100 mm min$^{-1}$ and the driving mechanism returned to the starting position after moving for 10 mm. After 10 or 30 shear cycles, CM or ABM-80 was removed carefully and the PBG which remained within circular region was collected and dried. The weight of the remaining PBG was recorded as $m_t$. The mass-loss ratio was calculated according to equation (2):

$$Mass\text{-}loss\ ratio = \frac{m_0 - m_t}{m_0} \times 100\% \quad (2)$$

***In vitro cytocompatibility tests***

Before the in vitro cytocompatibility tests, the adhesive hydrogels were purified in deionized water, air-dried overnight in blast drying oven at 40 °C, and then sterilized by ethylene oxide. The cytotoxicity of the as-obtained DAHs was measured by CCK-8 assay. The complete culture medium contained DMEM/F12 with 10% FBS and 1% penicillin-streptomycin. 20 mg of each as-obtained DAH sample (DAH-60, DAH-80 and DAH-100) was immersed in 1 mL complete culture medium at 37 °C for 72 h to prepare the conditioned culture medium. BMSCs were seeded in 24-well plates ($0.5\times10^4$ cells per well), treated with the conditioned culture media in experimental groups and with complete culture medium in blank control group (400 μL per well), and cultured at 37 °C. After 1, 3 and 7 days, 10% CCK-8 reagent was added into the wells and incubated for 45 min. The resulting solutions were transferred to 96-well plate. Then, the optical density (OD) at 450 nm was measured by a microplate reader (Epoch 2, BioTek, America).

Effect of DAHs on cell morphology was observed by fluorescence staining after 3 days of culture. Briefly, cells were fixed with 4% paraformaldehyde for 15 min, and permeabilized with 0.5% Triton X-100/PBS for 10 min. Then DAPI was used to stain cell nucleus, while Actin-Tracker Green was utilized to stain cytoskeleton. Finally, fluorescence images were taken using a confocal laser scanning microscope (FV3000, Olympus, Japan).

***In vitro osteogenesis assays***

Before the in vitro osteogenesis assays, the AH-80 was purified in deionized water, and then air-dried overnight in blast drying oven at 40 °C. The as-obtained DAH-80 and the control sample CM were sterilized by ethylene oxide. The osteogenic abilities of BMSCs cocultured with samples in cell culture insert (8 μm pore) were evaluated via ALP activity and calcium deposition. The ALP activity of cells was detected using an ALP Assay Kit at the $7^{th}$ and $14^{th}$ days of osteogenic induction. At each time point, cells were rinsed with PBS and lysed with 1% Triton X-100/PBS at 4 °C overnight, and then ALP activity was quantitatively measured. The formation of calcium deposits was detected using ARS staining. At the $14^{th}$ and $21^{st}$ days of osteogenic induction, cells were fixed with 4% paraformaldehyde and stained with ARS solution for 15 min, followed by calcium nodule observation. A semi-quantitative analysis of calcium deposits was determined with a microplate reader (Epoch 2, BioTek, America) at 562 nm.

***In vivo histocompatibility assays***

Before the in vivo histocompatibility assays, the AH-80 was purified in deionized water, and then air-dried overnight in blast drying oven at 40 °C. The as-obtained DAH-80 and the control sample CM were sterilized by ethylene oxide and immersed in PBS for 1 h for rehydration. 5-week-old male BALB/c nude mice (20–22 g weight) were used for evaluating the biocompatibility of DAH-80 in vivo under the permission of Animal Ethics Committee of Sun Yat-sen University (No. SYSU-IACUC-2022-000002). All mice were anaesthetized by injecting 2% pentobarbital sodium intraperitoneally (45 mg $kg^{-1}$). For each mouse, two skin incisions (10 mm length) were created on both sides of the back, and then a circular sample (6 mm diameter) was implanted subcutaneously on each side. After 14 or 28 days, the animals were euthanized. Tissues surrounding samples were excised and fixed with 4% paraformaldehyde for 48 h, followed by sequential alcohol dehydration. Tissue specimens were embedded in paraffin and cut into 4-μm sections. Sections were submitted for hematoxylin and eosin (HE) staining and immunohistochemical staining of CD68.

***In vivo assessments of bone augmentation abilities***

Before the in vivo augmentation ability tests, the AH-80 was purified in deionized water, integrated with CM and then air-dried overnight in blast drying oven at 40 °C. The as-obtained ABM-80 and the control sample CM were sterilized by ethylene oxide. 3-month-old male New Zealand white rabbits (2.0–2.5 kg weight) were used for in vivo experiment under the

permission of Animal Ethics Committee of Sun Yat-sen University (No. SYSU-IACUC-2021-000887, No. SYSU-IACUC-2023-000286). For each rabbit, after being anaesthetized by 3% pentobarbital sodium (30 mg $kg^{-1}$), the medial proximal tibia was exposed. A non-full-thickness circular defect was created with a trephine drill (6 mm diameter) and a round drill. Five nutrient foramina were then prepared with a pioneer drill to provide better blood supply for the bone graft materials. For the PBG groups, PBG was added in bone defect regions and then covered with ABM-80**,** while the same amount of PBG covered with CM and CM plus 4 pins (CM-4P) was set as negative control and positive control, respectively. For block bone graft (BBG) groups, the full-thickness circular autogenous BBG taken from the rabbit skull was grafted onto the defect region, and then covered with ABM-80, while the same size of BBG covered with CM was set as negative control.

After 10 days or 2 months of healing, animals were euthanized, and tibias were removed and fixed in 4% paraformaldehyde for 48 h. Bone defect regions were scanned by micro-CT (SkyScan 1276, Bruker, Germany). The CTvox software (v.3.3.0, Bruker, Germany) was used to implement 3D reconstructions and the CTAn software (v.1.20, Bruker, Germany) was used to analyze bone volume fraction (bone volume/total volume, BV/TV) quantitatively. The region of interest was defined as a cylinder (6 mm diameter and 2 mm height) from the plane formed by the upper edge of the defect cavity. Subsequently, the height of augmented bone was determined by the vertical height between the plane formed by the upper edge of the defect cavity and the top of the augmented bone.

After micro-CT scanning, all fixed tissues were immersed in EDTA decalcifying solution until specimens were decalcified completely. Specimens were then dehydrated, embedded into paraffin and cut into 4-µm sections. Sections were stained with HE and Masson's trichrome to exhibit the augmented bone. The as-obtained sections were observed via a digital pathology scanner (Aperio AT2, Leica, Germany).

***Statistical analysis***

Statistical analysis was carried out by SPSS software (IBM Corp, America). Data were expressed as mean ± standard deviation (SD) from at least three parallel experiments. For data with normal distribution and homogeneity of variance, statistical differences between groups were determined by Student's *t* test, one-way analysis of variance (ANOVA) followed by least significant difference (LSD) test. Data with abnormal distribution or heterogeneity of variance were analyzed by Mann-Whitney *U* test and Kruskal-Wallis's nonparametric test. $p < 0.05$ was considered statistically significant.

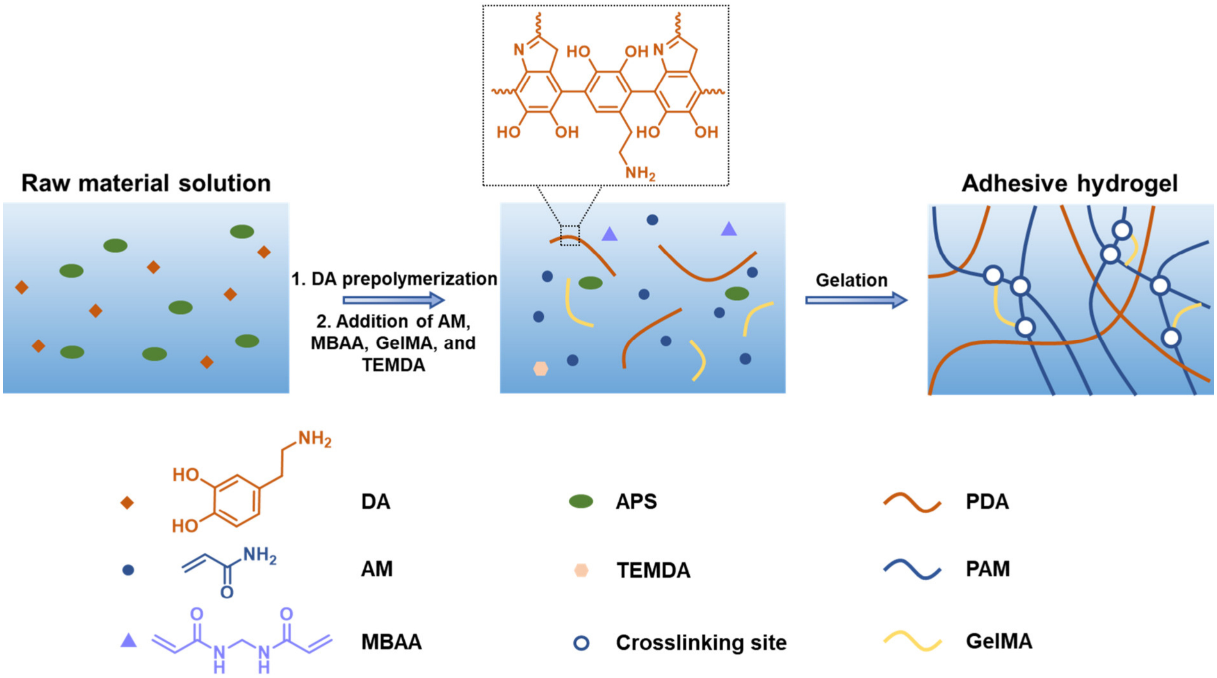


**Figure S1.** Schematic illustration for synthesis process of adhesive hydrogel.

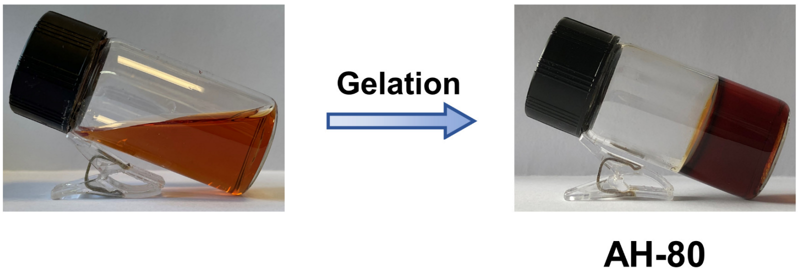


**Figure S2**. Digital photos of the gelation progress for AH-80.

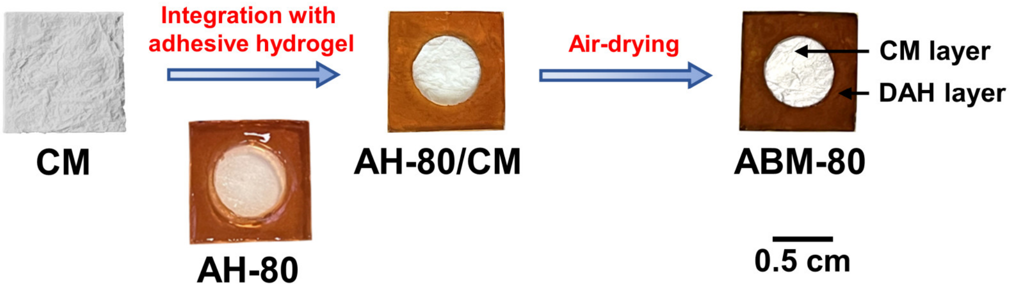


**Figure S3**. Digital photos of preparation procedures for ABM-80.

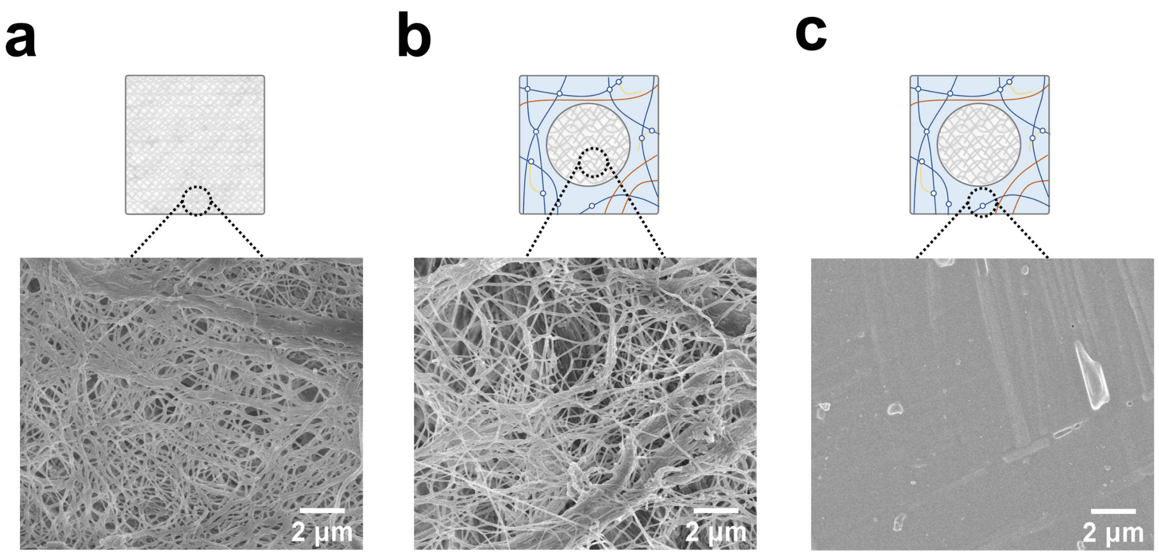


**Figure S4.** SEM images of a) the top-surface and b) bottom-surface of CM layer, and c) the surface of DAH layer of ABM-80.

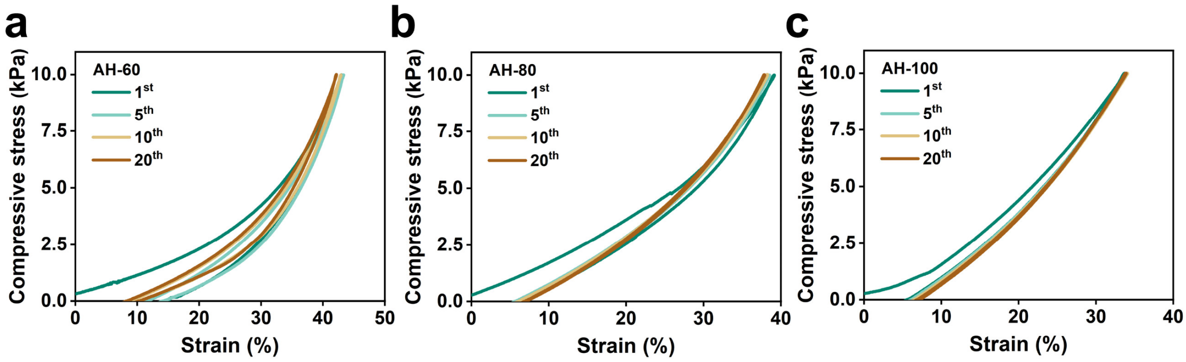


**Figure S5.** Cyclic compress loading-unloading curves of a) AH-60, b) AH-80, and c) AH-100.

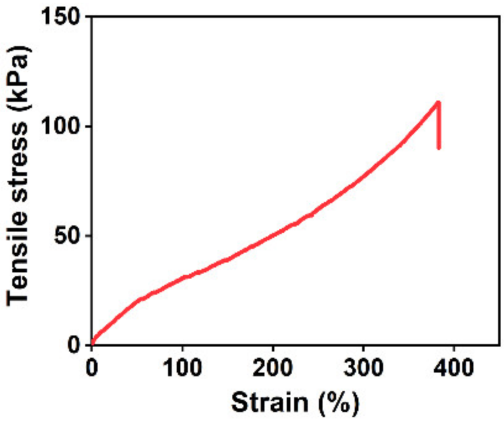


**Figure S6.** Tensile stress-strain curve of AH-80.

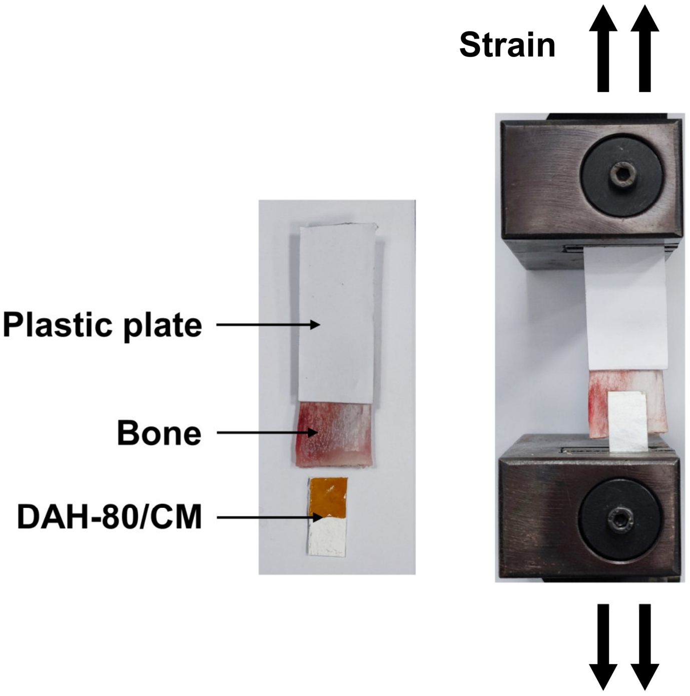


**Figure S7.** Measurement of adhesion between bone and DAH-80/CM.

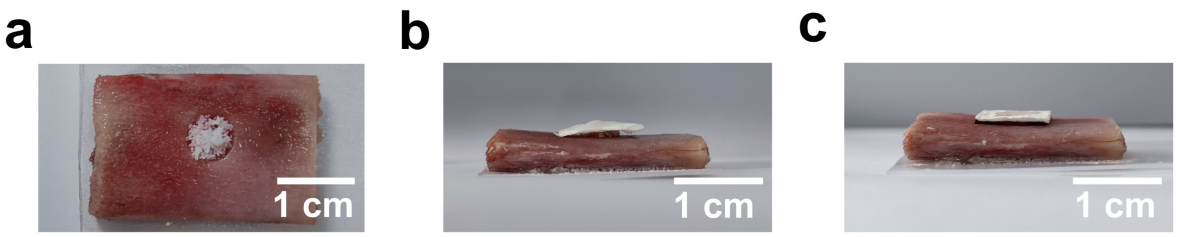


**Figure S8.** Digital photos of a) a circular bone defect filled with PBG, covered with b) CM or c) ABM-80.

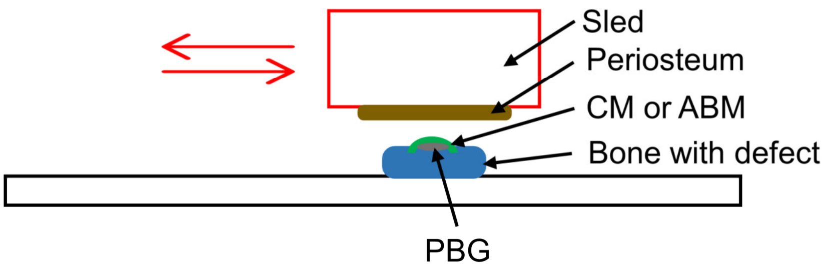


**Figure S9.** Schematic illustration of measurement of bone graft material leakage.

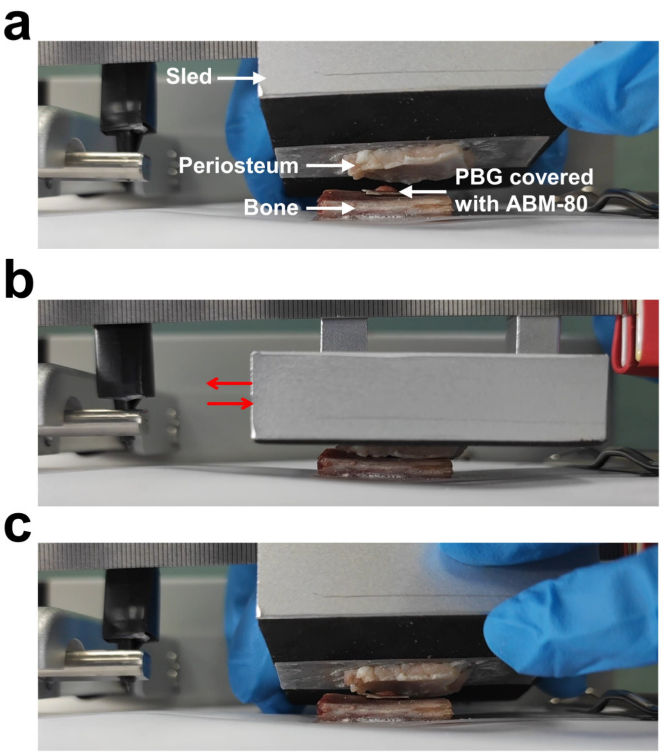


**Figure S10.** Digital photos of a) the measurement system of bone graft material leakage, b) the illustration of shear cycle, and c) the outstanding self-fixation and bone graft material immobilization effects of ABM-80 against shear.

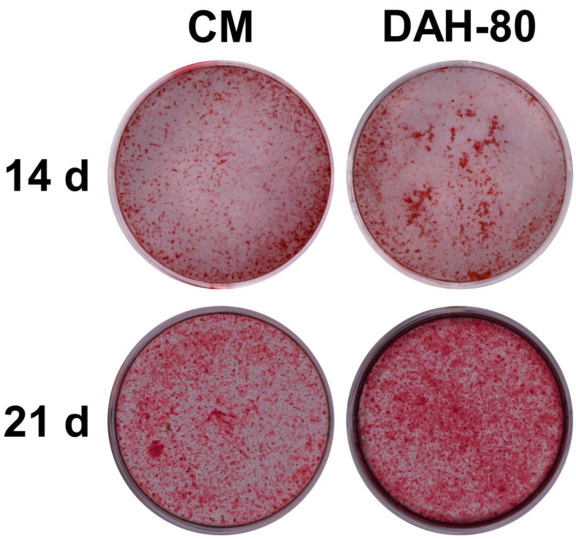


**Figure S11.** Representative images of ARS staining of BMSCs cocultured with CM or DAH-80 for 14 and 21 days in osteogenic induction medium (red for calcium nodule).

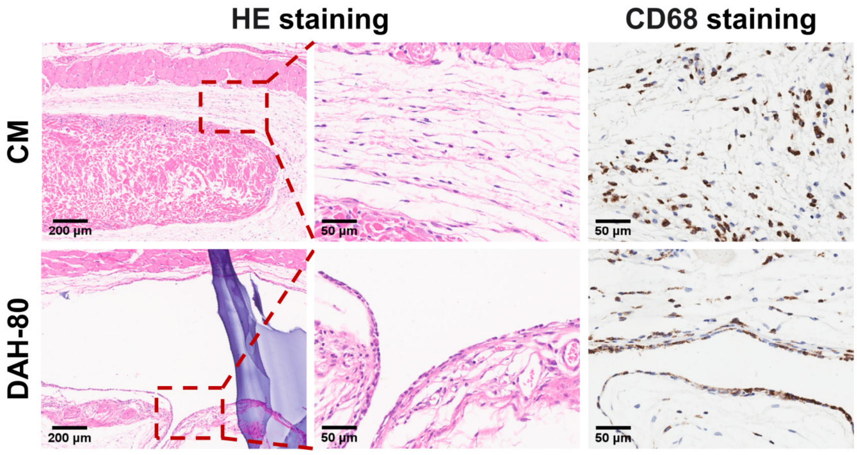


**Figure S12.** HE staining and CD68 immunohistochemical staining of tissues around CM and DAH-80 embedded in the backs of nude mice for 14 days.

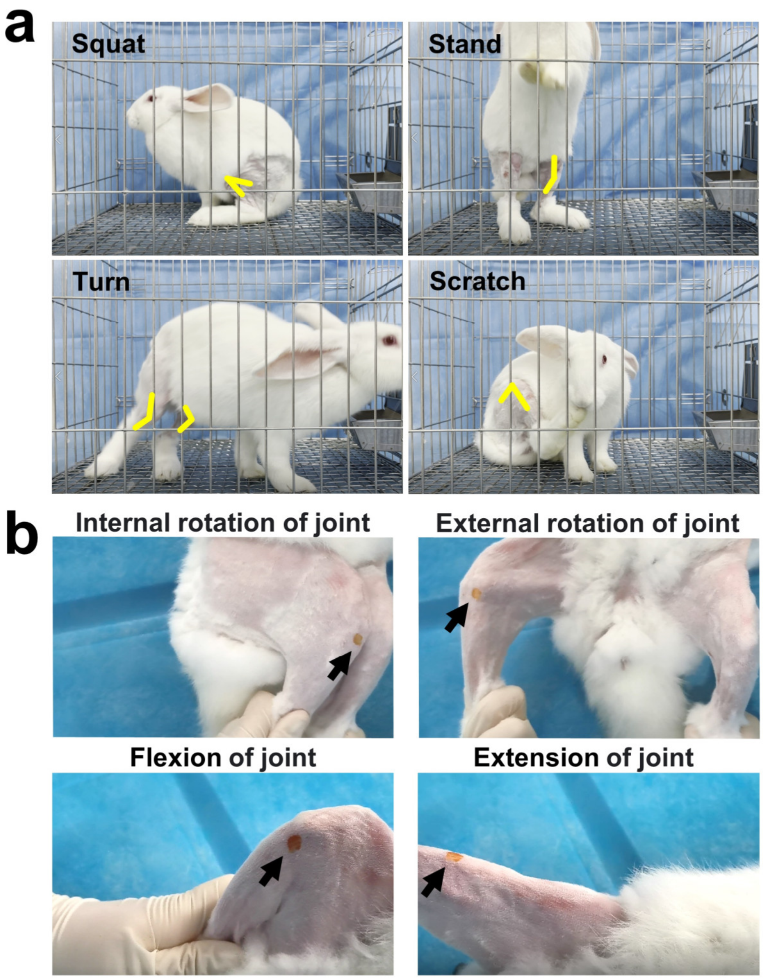


**Figure S13.** Digital photos of a) daily activities (yellow lines indicate the relative position of tibia and femur in daily activities), and b) knee joint movement modes of rabbit (the brown dot is a mark on the surface skin of the medial proximal tibia of a rabbit; black arrows indicate the position changes of the marked skin during knee joint movement).

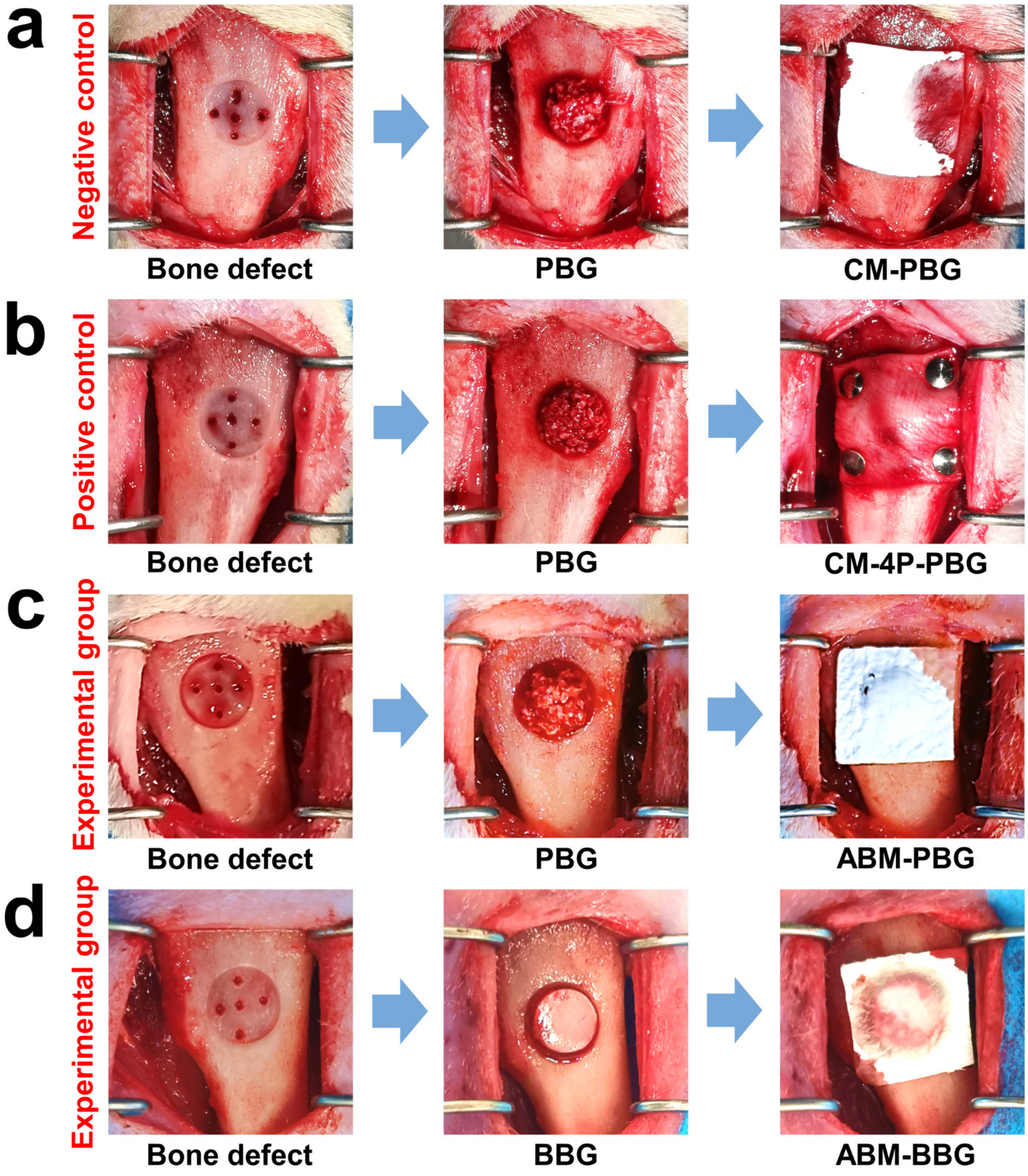


**Figure S14.** a-c) Digital photos of surgical procedures for the rabbit tibial defect models, including creating non-full-thickness circular defects, filling with PBG, and covering a) CM, b) CM-4P, or c) ABM. d) Digital photos of surgical procedures, including creating defects, filling with BBG, and covering with ABM.

**Table S1.** Synthesis of adhesive hydrogel samples.

| Sample | Raw material | | | | | | |
|---|---|---|---|---|---|---|---|
| | DA [g] | $H_2O$ [mL] | APS [g] | AM [g] | MBAA [mg] | GelMA [mg] | TEMDA [μL] |
| AH-60 | 0.15 | 30 | 1.05 | 7.50 | 45 | 30 | 56 |
| AH-80 | 0.15 | 30 | 1.05 | 7.50 | 60 | 15 | 56 |
| AH-100 | 0.15 | 30 | 1.05 | 7.50 | 75 | 0 | 56 |
